# Solvent Mixture Optimization in the Extraction of Bioactive Compounds and Antioxidant Activities from Garlic (*Allium sativum* L.)

**DOI:** 10.3390/molecules26196026

**Published:** 2021-10-04

**Authors:** Vytória Piscitelli Cavalcanti, Smail Aazza, Suzan Kelly Vilela Bertolucci, João Pedro Miranda Rocha, Adriane Duarte Coelho, Altino Júnior Mendes Oliveira, Laís Campelo Mendes, Maysa Mathias Alves Pereira, Ludmila Caproni Morais, Moacir Rossi Forim, Moacir Pasqual, Joyce Dória

**Affiliations:** 1 Department of Agriculture, Federal University of Lavras (UFLA), Lavras 37200-900, Brazil; vytoria.cavalcanti@estudante.ufla.br (V.P.C.); suzan@ufla.br (S.K.V.B.); jjoaomiranda7@gmail.com (J.P.M.R.); duartecoelho.adriane@gmail.com (A.D.C.); altinojrmendes@gmail.com (A.J.M.O.); lais.campelo21@gmail.com (L.C.M.); agro.maysa@gmail.com (M.M.A.P.); ludmilacaproni@gmail.com (L.C.M.); mpasqual@ufla.br (M.P.); 2 Laboratory of Phytochemistry, National Agency of Medicinal and Aromatic Plants (NAMAP/ANPMA), Taounate 34202, Morocco; aazzasmail@ymail.com; 3 Department of Chemistry, Federal University of São Carlos (UFSCar), São Carlos 13565-905, Brazil; mrforim@gmail.com

**Keywords:** extraction optimization, solvent mixtures, response surface methodology, phenols, thiosulfinates, antioxidants

## Abstract

Garlic is a health promoter that has important bioactive compounds. The bioactive extraction is an important step in the analysis of constituents present in plant preparations. The purpose of this study is to optimize the extraction with the best proportion of solvents to obtain total phenolic compounds (TPC) and thiosulfinates (TS) from dried garlic powder, and evaluate the antioxidant activities of the optimized extracts. A statistical mixture simplex axial design was used to evaluate the effect of solvents (water, ethanol, and acetone), as well as mixtures of these solvents, after two ultrasound extraction cycles of 15 min. Results showed that solvent mixtures with a high portion of water and pure water were efficient for TPC and TS recovery through this extraction procedure. According to the regression model computed, the most significant solvent mixtures to obtain high TPC and TS recovery from dried garlic powder are, respectively, the binary mixture with 75% water and 25% acetone and pure water. These optimized extracts presented oxygen radical absorbance capacity. Pure water was better for total antioxidant capacity, and the binary mixture of water–acetone (75:25) was better for DPPH scavenging activity. These optimized extracts can be used for industrial and research applications.

## 1. Introduction

In the last decades, there has been an increasing interest in natural antioxidants. Garlic plant (*Allium sativum* L.) and its extracts are used as food ingredients as well as health promoters [1]. Garlic cloves present phenolic and organosulfur compounds, as thiosulfinates, mainly related to antioxidant activity, having the potential for health promotion and contributing to protection against oxidative stress in humans [2,3]. Thus, garlic extracts have continually been used in herbal therapy.

In the state of the art, there are several studies that evaluated methods of extracting bioactive compounds from garlic, checking the extraction time, extraction temperature, particle size, and the best solvents, such as water, acetone, and ethanol [1,2,3,4,5]. However, there is a lack of information on what would be the best solvent mixture to obtain garlic extracts rich in bioactive compounds and antioxidant activity.

Extraction is the initial and the most critical step in isolating different types of bioactive compounds [6], solvent extraction being one of the most commonly used to recover antioxidant compounds from plants [7]. In general, optimization studies evaluate the effects of one independent variable (such as the solvent) on a response variable (such as the content of bioactive compounds extracted from the plant by the solvent) over time to achieve maximum benefits. However, this is not sufficient for finding associations between multiple independent variables (such as finding the proportion of each solvent in a solvent mixture to improve the extraction process), thereby necessitating alternative multivariate statistical analyses to achieve these results.

The last few years have highlighted the use and importance of the design of experiments (DOE) in process modeling in food science and technology, and the DOE followed by multiple regression analysis is the so-called response surface methodology (RSM) [8], widely used for extraction optimization. Through the RSM it is possible to determine the best solvent mixture without testing a high number of solvent proportions. Instead, known proportions of solvent in a specific DOE (such as simplex-centroid design or simplex axial design) are used to estimate the best solvent mixture by multiple regression analysis, consequently reducing the benchwork and the amount of solvent used.

**Figure 1:**
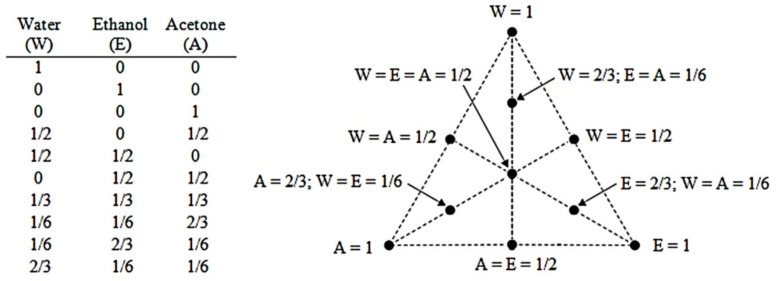
Illustration representing the mixture design simplex axial design (SAD). W—water, E—ethanol, A—acetone.

In the simplex-centroid design, the different conditions tested formed a triangle, with pure components in the vertex representing 100% of each solvent. The middle points on each side represent permutations of the binary blends (1/2:1/2:0; 1/2:0:1/2; 0:1/2:1/2), and the center point represents a ternary mixture (1:1:1). This design is sometimes augmented with interior points (axial points) representing 2/3 of one of the solvents and 1/6 of the others (Figure 1) and is also known as a simpex axial design (SAD) [9].

In this sense, the present investigation identified the most appropriate solvent mixture for thiosulfinates and phenolic compounds extractions from garlic, by using a simplex axial design, and confirmed the antioxidant activity of the optimized extracts. These solvent-mixture optimized extracts can be used for industrial and research applications, presenting a high number of bioactive compounds and, consequently, high antioxidant activities.

## 2. Materials and Methods

### 2.1. Chemicals and Standards

The standard gallic acid (98.0%) was purchased from Vetec Químimca Fina (Rio de Janeiro, RJ, Brazil) ascorbic acid (99.7–100.5%), ferulic acid (>99%), allicin (S-Allyl 2-propene-1-sulfinothioate), diallyl disulfide (≥98%), and Trolox (≥98%) were purchased from Sigma-Aldrich (St. Louis, MO, USA). Ethanol (99.8%), Folin–Ciocalteu reagent, sodium carbonate, 5, 5′-dithiobis (2-nitrobenzoic acid) (DTNB), sulfuric acid, sodium phosphate, 2, 2′-Azobis (2-methylpropionamidine) dihydrochloride (AAPH), 2, 2-Diphenyl-1-picrylhydrazyl (DPPH), and Hepes buffer were purchased from Sigma-Aldrich. Acetone (99.5%), anhydrous bibasic sodium phosphate (P.A.-A.C.S.), and monobasic sodium phosphate (P.A.-A.C.S.) were purchased from Êxodo científica (Hortolândia, SP, Brazil), ammonium molybdate and fluorescein from *Synth* (Diadema, SP, Brazil), and cysteine from Vetec Químimca Fina (Rio de Janeiro, RJ, Brazil). HPLC grade methanol was purchased from AppliChem Panreac^®^.

### 2.2. Samples

Garlic samples (cultivar Gigante Roxo) were obtained from the 2016/2017 crop of a producer in Inconfidentes, MG, Brazil. Garlic cloves were dried in a ventilated oven at 40 °C until they reached a constant weight. They were then ground in a household food grinder.

### 2.3. Extraction Procedure

The extractions were performed in triplicate according to the following procedure: 50 mg dried garlic cloves were submitted to ultrasound-assisted extraction (NOVA Instruments, NI 1204, Piracicaba, SP, Brazil) at room temperature and frequency of 60 Hz, with 1 mL of solvent mixture (the details are described in Section 2.4). The extraction was carried out through two ultrasound-assisted extraction cycles of 15 min, renewing the solvent after each cycle. During each ultrasound bath cycle of 15 min, the samples were vortex-agitated for 1 min three times (after each 5 min). After each ultrasound cycle, the extracts were centrifuged at 12,000 rpm (9676.8× *g*) (Thermo Fisher Scientific, Heraeus Pico 17, Waltham, MA, USA) for 15 min at room temperature, supernatants were recovered and stored at 4 °C.

### 2.4. Evaluation of Solvent Effects by Simplex Axial Design (SAD)

To optimize the extraction process, the SAD was chosen because of the development of the axial points (2/3:1/6:1/6; 1/6:2/3:1/6; 1/6:1/6:2/3). In addition to the pure solvent in the vertex (1:0:0; 0:1:0; 0:0:1), the middle points on each side (1/2:1/2:0; 1/2:0:1/2; 0:1/2:1/2), and the center point (1:1:1) (Figure 1). This DOE provides increasing the accuracy in the analysis of the proportions of each solvent (water (W), ethanol (E), and acetone (A), as shown in Figure 1) to be used in the solvent mixture to the recovery of thiosulfinates and total phenolic compounds from dried garlic cloves. Each extraction with the solvent mixture or with the pure solvents was done with three replicates.

### 2.5. Total Phenolic Content (TPC)

A modification of the Folin–Ciocalteu method [10] determined the total phenolic content as follows: 40 μL extract was mixed with 120 μL Folin–Ciocalteu reagent (0.2 N) in 96-well microplates for 5 min, and then 120 μL sodium carbonate solution (75 g L^−1^) was added. This mixture was incubated at room temperature under dark conditions for 2 h, and their absorbance was read at 760 nm in a microplate reader TECAN Infinity^®^ M200 PRO (Tecan Group Ltd., Männedorf, Zürich, Switzerland) operated by Icontrol^®^ software version 3.37 (Tecan Group Ltd.). The concentration of the calibration curve (*y* = 68.839*x* + 0.0553, R² = 0.999) ranged from 8.0 × 10^−3^ to 1.0 mg mL^−1^ in an ethanolic solution of gallic acid. The experiment was conducted in triplicate, and the results were expressed as mg gallic acid equivalents (GAE) g^−1^ dry weight (DW).

### 2.6. Thiosulfinates (TS)

The quantitative determination of total thiosulfinates in the extracts was carried out following the methodology described by Li and Xu [11] with some modifications and was adapted to be performed directly on the microplate, as described below.

Extracts had been dried in the oven at 40 °C and re-solubilized in water. The solution of cysteine (20 mmol L^−1^) was freshly prepared in 50 mmol of L^−1^ Hepes buffer (pH 7.5). The concentration of cysteine was determined by measuring the amount of 2-nitro-5-thiobenzoate (NTB) formed after reaction with 5, 5′-dithiobis (2-nitrobenzoic acid) (DTNB). All of the reactions were carried out at room temperature. The cysteine solution was diluted 20 times, and 50 μL was added to the microplate, with 50 μL of the extracts or water (negative control), and the mixture was incubated for 15 min. This reaction mixture was diluted 2 times by adding 100 μL of distilled water. Then, 100 μL of 50 mmol L^−1^ Hepes buffer (pH 7.5) containing 1.5 mmol L^−1^ DTNB was added, and the absorbance was measured at 412 nm after 15 min. Finally, the thiosulfinate concentrations were determined by the following equation:

(1) $$C_{thiosulfinates}\left(mmol\;mL^{-1}\right)=\frac{\left(\Delta A_{412}\times 2\right)}{\left(2\times 14150\right)}$$

where Δ*A*_412_ = *A*_0_ − *A*. *A*_0_: absorbance of the negative control, *A*: the absorbance of the reaction with the extract. Δ*A*_412_ was multiplied by 2 due to the dilution made in the reaction mixture, 14150 is the molar extinction coefficient of NTB, and 2 means that a half amount of cysteine reduced denotes the number of thiosulfinates. The experiment was performed in triplicate, and the results were expressed as µmol g^−1^ DW.

### 2.7. HPLC Analysis

An Agilent 1200 Series (Agilent Technologies^®^, Waldbronn, Germany) HPLC system composed of a quaternary pump (G1311A), degasser system (G13222A), ALS autosampler (G1322A), diode array detector (G1315D), and data acquisition system operated by OpenLAB software was employed. The analyses were performed on a reversed-phase column (ZORBAX Eclipse XDB-C18, 4.6 × 150 mm, 5 μm, Agilent, USA) in combination with a guard column (Eclipse XDB-C18, 4.6 × 12.5 mm, 5 μm, Agilent, USA).

The preparation of the samples for HPLC was performed as described below: 1 mL of the extract was rotary evaporated to remove the solvent. Then, 1 mL of HPLC grade methanol 50% was added to the dried extract, directly in the glass balloon. The extract was solubilized using an ultrasound bath for 2 min and then centrifuged at 10,000 rpm (8064ϗ *g*) for 10 min. The supernatant was collected in a vial and 10 μL of this sample was automatically injected.

The chromatographic conditions were kept at a flow rate of 1.0 mL/min and a column temperature of 40 °C, and the peaks were simultaneously identified using UV absorbance at 210 nm. Methanol and water were employed as the mobile phase in a gradient elution as described in Table 1. Phosphoric acid 0.1% was added to the eluents. The injections were performed in triplicate and the injection volume was 10 µL for each replicate. The chromatographic peaks of the analytes were confirmed by comparing their retention time and UV spectra with those of the reference compounds. Allicin and diallyl disulfide were used as reference compounds for organosulfur compounds. Ferulic acid was used as a reference compound for phenolic compounds.

### 2.8. Antioxidant Activities

#### 2.8.1. Total Antioxidant Capacity (TAC)

According to Prieto, Pineda, and Aguilar, the extracts’ TAC was evaluated by green phosphomolybdenum complex formation [12]. An aliquot of 25 µL of the extract solution was combined with a 1 mL reagent solution (0.6 mol L^−1^ sulfuric acid, 28 mmol L^−1^ sodium phosphate, and 4 mmol L^−1^ ammonium molybdate) in Falcon 15 mL tubes. The Falcon tubes were capped and incubated in a water bath at 95 °C for 90 min. After the mixture cooled to room temperature, the absorbance was measured at 695 nm against a blank. The aqueous ascorbic acid solution calibration curve (*y* = 14.535*x* − 0.0065, R² = 0.996) comprised a concentration range from 3.9 × 10^−3^ to 5.0 mg mL^−1^. The experiment was conducted in triplicate, and the results (ascorbic acid equivalent antioxidant activity) are the mean values expressed as g of ascorbic acid equivalents (AAE) g^−1^ DW.

#### 2.8.2. DPPH Free Radical Scavenging Activity

The optimized extracts were tested for the scavenging effect on DPPH (2, 2-diphenyl-1-picrylhydrazyl) radical according to the method of Soler-Rivas, Espín, and Wichers [13]. Each sample in different concentrations (100 µL) and 500 µL of a 60 µmol L^−1^ ethanolic solution of DPPH was then added to 1.5 mL microtubes and vortexed. After 60 min of reaction at room temperature, the tubes were centrifuged for 10 min at 5000 rpm. Finally, the reactions were placed in a 96 well microplate and absorbance measurements were made at 517 nm.

The percentage of inhibition was calculated using the following equation:

(2) $$\mathrm{Inhibition}\;\left(\%\right)=\left[\frac{\left(A_{0}-A_{1}\right)}{A_{0}}\right]\times 100$$

where *A_0_* is the absorbance of the control (without sample) and *A_1_* is the absorbance in the presence of the sample. The sample concentration in which the inhibition percentage reaches 50% (IC_50_) was obtained by plotting the inhibition percentage against extract concentrations and the IC_50_ values were expressed as mg DW mL^−1^. Tests were carried out in triplicate.

#### 2.8.3. Oxygen Radical Absorbance Capacity (ORAC)

The ORAC assay was carried out in a microplate reader (TECAN Infinity^®^ M200 PRO, operate by Icontrol^®^ software version 3.37), and the procedure was based on a previous report by Ou, Hampsch-Woodill, and Prior [14]. Extracts had been dried in the oven at 40 °C and re-solubilized in 75 mmol L^−1^ phosphate buffer (pH 7.4). All reagents were prepared in a 75 mmol L^−1^ phosphate buffer (pH 7.4). In the final assay mixture (0.210 mL total volume), 30 μL of samples were placed in a 96 well microplate with 130 μL of fluorescein (FL) (6.3 × 10^−8^ M), representing the target of the free radical attack, and were pre-incubated at 37 °C during 10 min. Then, 30 μL of AAPH (1.28 × 10^−2^ M) was added as a peroxyl radical generator. Trolox was used as the control standard. The analyzer was programmed to record the fluorescence of FL every minute for 150 min, shaking before each reading after the addition of AAPH. Results were calculated based on differences in the areas under the sodium fluorescein decay curve (AUC) between the blank and a sample. The AUC was calculated as:

(3) $$\mathrm{AUC}=1+\frac{f_{1}}{f_{0}}+\frac{f_{2}}{f_{0}}+\frac{f_{3}}{f_{0}}+\frac{f_{4}}{f_{0}}+\left[\ldots \right]+\frac{f_{150}}{f_{0}}$$

where *f_0_* is the initial fluorescence reading at 0 min and *fn* is the fluorescence reading at time *n*. The Trolox calibration curve (*y* = (1.0 × 10^9^) *x* + 6.0 × 10^6^, R² = 0.997) comprised a concentration range from 0.25 to 0.0019 mg mL^−1^ and results were expressed as µmol Trolox equivalent (TE) g^−1^ DW. Dynamic curves from an ORAC assay were plotted using the free version of GraphPad Prism version 9.2.0 (GraphPad Software, LLC, San Diego, CA, USA). Tests were carried out in triplicate.

### 2.9. Statistical Analysis

All of the analyses above were carried out in triplicate and values expressed as mean ± standard deviation. Analysis of variance (ANOVA) was applied to determine the fit of the multiple regression model (*p* < 0.05) to evaluate the significant effects of the variables and the interactions between them. The response surface and contour graphs of the model were generated from the regression coefficients. The data of the antioxidant activities were submitted to analysis of variance, and the means were compared by Tukey’s. The analyses were performed using the free trial of STATISTICA version 14.0.0.15 software (TIBCO Software Inc., Palo Alto, CA, USA) [15].

## 3. Results and Discussion

### 3.1. Solvent Mixture Composition in TPC and TS Extraction

Simplex axial design and response surface methodology were used to evaluate how the solvent mixture affected polyphenol and thiosulfinate recovery from garlic (A. sativum), just like Strati et al. [16], who already applied the response surface methodology effectively to optimize the phenolic extraction from Allium ampeloprasum.

Observing the effects of the composition of the solvent on TPC and TS content in garlic cloves, mixtures with a high portion of water exhibited high amounts of these bioactive compounds (Table 2). This high TPC amount can occur due to the wide range of phenols that the aqueous mixtures can dissolve, as well as increased solvation provided by water [17]. Additionally, for thiosulfinates, most of the articles present extraction with pure water [11,18,19].

The highest TPC recovery occurred in the extractions using a ternary mixture with a higher portion of water (water–ethanol–acetone, 2/3:1/6:1/6, *v*/*v*/*v*), binary mixture with water and acetone (1:1, *v*/*v*), pure water, and binary mixture with water and ethanol (1:1, *v*/*v*). The lowest TPC recovery was observed in the extraction with binary mixture with ethanol and acetone (1:1, *v*/*v*), pure acetone, and pure ethanol (Table 2). The highest TS recovery occurred in the extractions using pure water, ternary mixture with a higher portion of water (water–ethanol–acetone, 2/3:1/6:1/6, *v*/*v*/*v*), and binary mixture of with water and acetone (1:1, *v*/*v*). The lowest TS recovery occurred in extraction with the binary mixture with ethanol and acetone (1:1, *v*/*v*) (Table 2). Those differences were due to the difference in the extraction solvents’ polarities, which might influence the solubility of the chemical constituents in a sample and its extraction yield [20]. This knowledge highlights the importance of different test proportions of each solvent, which have different polarities, in the extraction process to find the solvent mixture that can extract the highest number of bioactive compounds.

The increase of water portions in solvent mixtures increases TPC and TS in *A. sativum* extracts (Table 2). In contrast, Tomšik et al. [21] observed that an increase of ethanol concentration to 70% presented high bioactive compound concentrations from *Allium ursinum* extracts; however, they used a temperature of 80 °C, concluding that it had a notable influence. Temperature increases can modify the physicochemical properties of water, making it similar to the properties of organic solvents, which can improve the solubility of organic molecules [22]. The combined use of a high portion of water with small amounts of an organic solvent may increase the phenolic compound’s extraction and thiosulfinates stability because of the polar medium created and facilitate the extraction of molecules that have an affinity with organic solvents [23,24].

### 3.2. Mixture Designs

#### 3.2.1. Total Phenolic Compounds (TPC)

**Figure 2:**
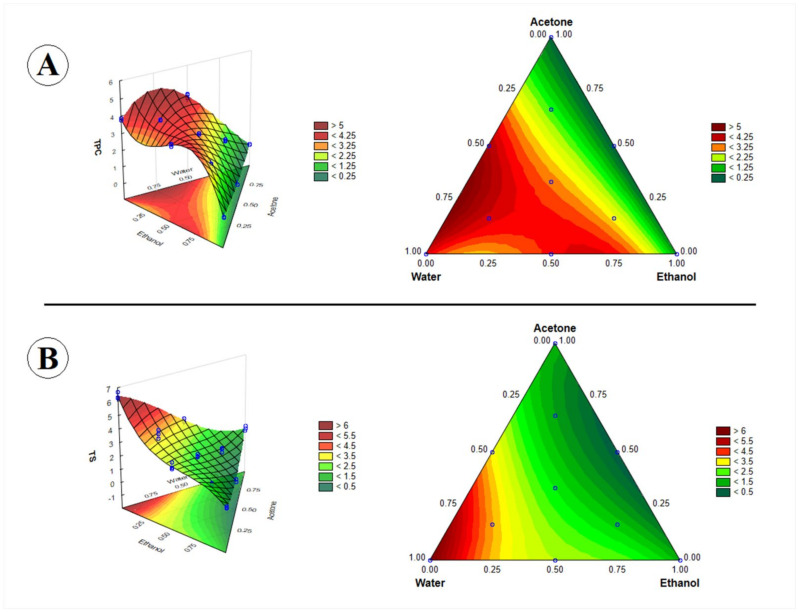
Cubic response surface and contour plot showing the effects of solvent mixture on TPC and TS content. (**A**) total phenolic compound (TPC) content (mg GAE g^−1^ DW); (**B**) thiosulfinate (TS) content (μmol g^−1^ DW).

Water, or alcohol and acetone with different amounts of water, are important solvents used to extract phenolic compounds from plant materials [25]. In this study, we have determined the optimum values of the independent variables (water, ethanol, and acetone) to achieve the maximum response extract total phenolic compounds from *A. sativum* dried cloves. The response surfaces and contour plots obtained for TPC by mixture design as a function of the percentage composition of water, ethanol, and acetone are illustrated in Figure 2.

ANOVA was applied, showing a fit to the linear, quadratic, and cubic models, with R² of 0.729, 0.972, and 0.998, respectively. Thus, the cubic model was the best to predict the behavior of the mixture and is given by the equation correlating the three variables and the analytical response as follows:

(4) $$\begin{array}{l} \mathrm{TPC}=+3.8107171395101\times W+0.83710763138243\times E+0.34927947471515\times A\\ \;+5.1728373167659\times W\times E+8.7652479819436\times W\times A-1.3389501705793\times E\times A\\ \;+2.5355321745271\times W\times E\times A-11.942432941375\times W\times E\times \left(W-E\right)\\ \;+5.2581859589796\times W\times A\times \left(W-A\right)+0 \end{array}$$

The analysis of the main effects and their interactions in the form of analysis of variance (ANOVA) are presented in Table 3 at the 95% confidence level (*p* < 0.05). The cubic model for TPC content showed no significant lack of fit at the 95% confidence level (Table 3), thereby confirming the model validity and indicating that the model developed is reliable and accurate to predict the relevant responses [26].

The highest values of TPC on the response surface and contour graph (Figure 2) occurred between the treatments with water (100%), water/ethanol/acetone (2/3:1/6:1/6), and water/acetone (1/2:1/2). The proportions optimized according to the response surface as being the maximum inside the experimental domain were water (75%), acetone (25%), and ethanol (0%). Kiassos, Mylonaki, Makris, and Kefalas [27] found a higher total polyphenol content in the extractions with the highest alcohol concentration evaluated (60%) for *Allium cepa*; however, they did not test pure water and had their lowest ethanol concentration at 40%, presenting the importance of organic solvents in the extraction of TPC.

**Figure 3:**
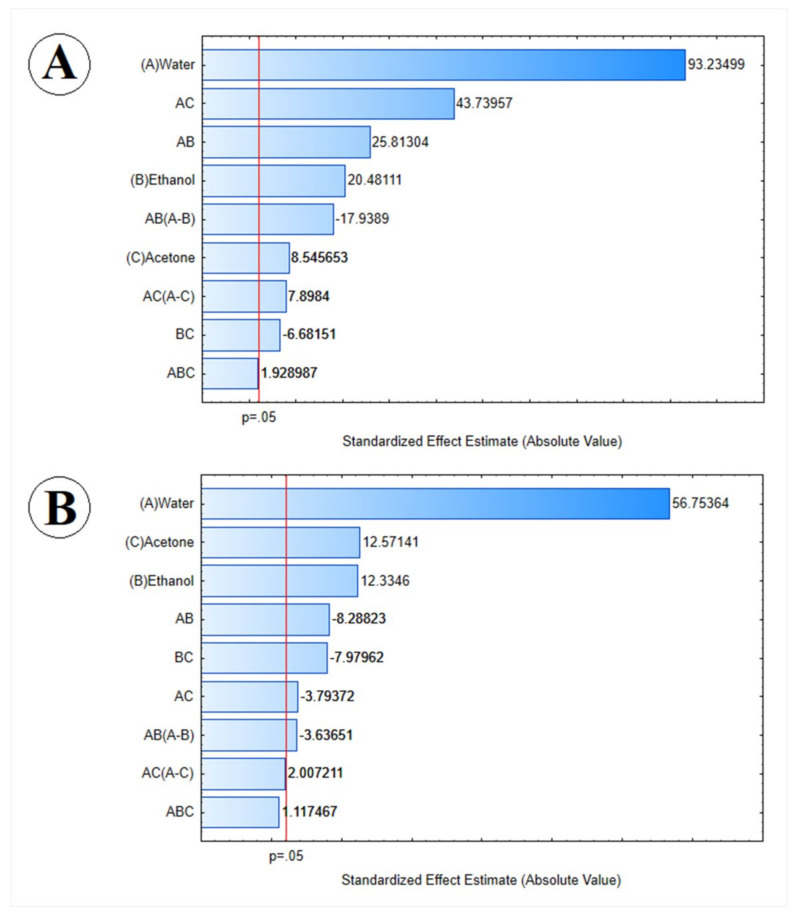
Standardized (*p* <  0.05) Pareto chart representing the estimated effects of parameters and parameter interactions on (**A**) total phenolic compound and (**B**) thiosulfinate recovery.

To examine the relative importance of the main effects and their interactions with statistical significance (*p*  <  0.05), a standardized Pareto chart (Figure 3) was employed. Between the factors that were significant at the level of 0.05, which extended beyond the reference line, water (A) and the interaction between water and acetone (AC) were the factors that presented a higher standardized effect in the extraction of TPC from dried garlic cloves.

Water (A) and its interaction with acetone (AC) presented the most significant effect on phenolic compound extraction (Figure 3). Maximum total phenol content can be expected for mixtures mostly rich in water. Rajha et al. [28] mentioned that water contributes to the solute desorption from the matrix and ethanol improves its solubility, using solvent mixtures a critical variable that can improve the extraction rates. Do et al. [23] also observed that the TPC extraction yield increased when it increased the solvent mixture’s water proportion. For TPC extraction in *Limnophila aromatica*, the mixtures with organic solvents, such as ethanol, methanol, and acetone, presented better extraction yields than pure solvents [23], as shown in our study as well.

#### 3.2.2. Thiosulfinates (TS)

The highest amounts of TS were observed in pure water, followed by ternary solvent mixture containing water–ethanol–acetone (2/3:1/6:1/6, *v*/*v*/*v*), and binary mixture with water and acetone (1:1, *v*/*v*) (Table 2).

The ANOVA test showed a fit to the linear, quadratic, and cubic models, with R² of 0.913, 0.983, and 0.990, respectively, and the cubic model was selected to explain the behavior of the mixture and is given by the equation correlating the three variables and the analytical response as follows:

(5) $$\begin{array}{l} \mathrm{TS}=+6.4097045898843\times W+1.3930588767909\times E+1.4198031994611\times A\\ \;-4.589528069537\times W\times E-2.1007371003208\times W\times A-4.4186366944686\times E\times A\\ \;+4.0587362672094\times W\times E\times A-6.6895604673989\times W\times E\times \left(W-E\right)\\ \;+3.6923801304256\times W\times A\times \left(W-A\right)+0 \end{array}$$

The cubic model for thiosulfinate content showed no significant lack of fit at the 95% confidence level (Table 3), thereby confirming the model validity.

The highest TS content value on the response surface and contour graph (Figure 2) was seen to occur in the vertex of water (100%). The TS content increase achieved the maximum at 100% of water and started to decrease slightly when the water concentration decreased (Figure 2); thus, the solvent optimized to the thiosulfinate extract tended to be pure water, which was the maximum inside the experimental domain. The results illustrated in Figure 3 confirm that water (A) was the most influential parameter at the 95% confidence level.

Cañizares et al. [29] carried out the extraction of garlic with different solvents, presenting a higher extractive yield with acetone. They justified that this solvent can degrade the cellular walls of vegetables, increasing their permeability and increasing their extractive power. Cañizares et al. [30] also used acetone for the extraction of thiosulfinates in garlic. In this work, pure acetone did not present the highest contents of thiosulfinates in garlic extracts. The presence of acetone in the mixtures with high amounts of water did show better results than pure acetone. However, the best solvent to extract thiosulfinates was found to be pure water. It is important to highlight that the use of water instead of organic solvents has the advantage of being greener, i.e., it does not generate waste and is compatible with the food and pharmaceutical industry.

### 3.3. Chromatographic Profile of Garlic Optimized Extracts

**Figure 4:**
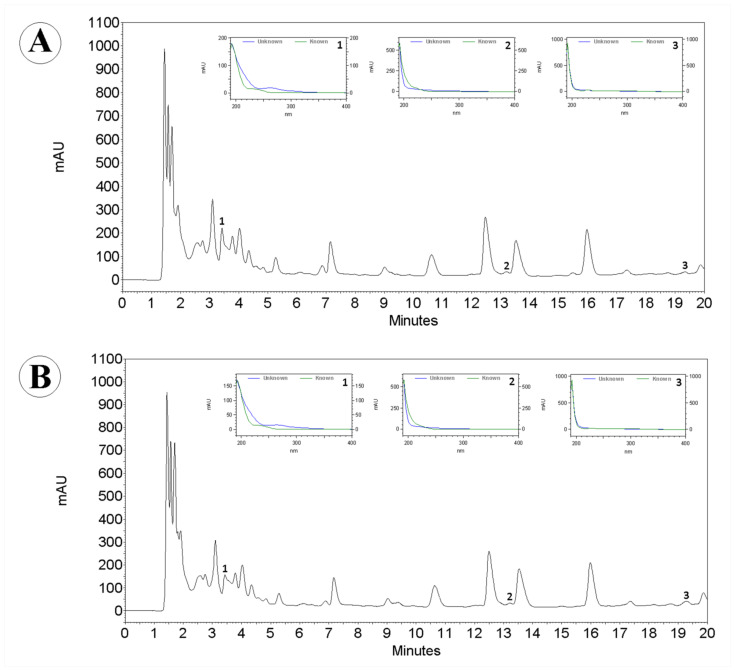
HPLC profiles obtained for garlic extracts using 100% water (**A**) and a binary mixture with 75% water and 25% acetone (**B**) as the solvent and UV spectra recorded online by DAD in the peaks from diallyl disulfide (1), allicin (2), and ferulic acid (3).

The chromatographic analyses of garlic optimized extracts (100% water and 75% water with 25% acetone) was performed to identify the presence of organosulfur compounds and phenolic compounds. Allicin and diallyl disulfide were used as reference compounds for organosulfur compounds. Ferulic acid was used as a reference compound for phenolic compounds. HPLC fingerprints registered at 210 nm for the optimized extracts indicated the presence of these bioactive compounds by comparison with the retention time (RT) and the UV spectra of reference compounds: diallyl disulfide (**1**, RT = 3.37 min), allicin (**2**, RT = 12.76 min), and ferulic acid (**3**, RT = 19.07 min) (Figure 4).

The optimized extracts showed similar HPLC profiles with minor differences, and both presented diallyl disulfide, allicin, and ferulic acid in their composition (Figure 4). Previous studies confirmed the presence of allicin and diallyl disulfide [5,31,32,33] between the organosulfur constituents, and the presence of ferulic acid [32,33,34,35] as one of the major phenolic acids in garlic extracts.

Allicin is one of the main active constituents in garlic [36,37], but it is highly unstable and is quickly decomposed in other stable organosulfur constituents, such as diallyl disulfide [33]. Alrumaihi [38] highlighted that allicin and diallyl disulfide are important active compounds that modulate several biological activities and help prevent pathogenesis.

The confirmation of the presence of these constituents indicates that these optimized extracts, with pure water (100%) and with 75% water and 25% acetone, will exhibit biological activities such as antioxidant activity. Antioxidant activity has received a lot of attention due to the various health problems triggered by oxidative stress [39]. In this context, obtaining extracts rich in antioxidants is of interest.

### 3.4. Antioxidant Activities

In order to estimate the antioxidant activity of the optimized extracts, molybdenum-reducing antioxidant potential (total antioxidant capacity-TAC), DPPH free radical scavenging activity (DPPH IC50), and oxygen radical absorbance capacity (ORAC) were evaluated. Phenolic compounds and thiosulfinates are known for their good antioxidant activity [40,41]. Both optimized extracts for TPC and TS showed results for all of these antioxidant activities (Table 4).

There are no significant differences between the extracts in the oxygen radical absorbance capacity (ORAC, *p* = 0.2979). We found ORAC results of 794.63 and 882.47 µmol TE g^−1^ DW, respectively, for garlic extracts with the binary mixture of 75% acetone and 25% water and with pure water (100%) as solvent. Beretta et al. [42] found an ORAC of 13.435 µmol TE g^−1^ for fresh garlic aqueous extract. Morales-Soto et al. [43] showed ORAC ranging from 8.32 to 43.08 µmol TE g^−1^ for garlic extracts using the binary mixture of 80% methanol and 20% water as solvent. Guizellini et al. [44] observed ORAC of 107.5 µM TE g^−1^ in their garlic extract using 70% methanol and 30% water as solvent. These results found in the literature confirm that extracts with pure water and with binary mixtures of water and organic solvents provide extracts with oxygen radical absorbance capacity (ORAC).

Pure water exhibited the best result (200.31 mg AAE g^−1^ DW) in the total antioxidant capacity (TAC, *p* = 0.0001) (Table 4). Denre et al. [45] also observed the molybdenum-reducing antioxidant potential for garlic cultivars aqueous extract, presenting values between 5.93 and 13.47 mg AAE g^−1^.

The binary mixture of 75% water and 25% acetone was more efficient in DPPH (*p* = 0.0175) free radical scavenging activity, with lower IC_50_ (2.88 mg mL^−1^) than pure water (3.40 ± 0.35 mg mL^−1^) (Table 4). Škrovánková et al. [46] showed IC_50_ ranged from 163.4 to 177.5 mg mL^−1^ for garlic dry commercial products extracted with methanol (80% *v*/*v*). Denre et al. [45] found DPPH IC_50_ ranged from 3.28 to 4.04 mg mL^−1^ for garlic cultivars aqueous extracts, presenting similar results to those found in the optimized extract with pure water (3.40 mg mL^−1^). All of these results confirm that extracts with binary mixtures of water and organic solvents provide extracts with better DPPH free radical scavenging activity than extracts with pure water.

These results also confirm the relationship between the amount of the bioactive compounds (TPC and TS) and the antioxidant activity of garlic cloves, reported by several authors [40,42,44,47]. Beretta et al. [42] also observed antioxidant activity in six Allium vegetable species aqueous extract containing thiosulfinates and phenols.

This research presented the importance of using a small portion of acetone in a high amount of water as the solvent mixture for better extraction of phenolics, and the use of pure water for better extraction of thiosulfinates from garlic cloves. Obtaining garlic extracts with high content of phenolics and thiosulfinates, consequently with high antioxidant activities, is of high interest for the food and pharmaceutical industry and the researchers that want to evaluate the effect of different treatments on garlic’s phenolic and thiosulfinate content.

## 4. Conclusions

The use of pure water, binary mixtures of water with ethanol (1:1, *v*/*v*) or acetone (1:1, *v*/*v*), and ternary mixture with high proportions of water (water–ethanol–acetone, 2/3:1/6:1/6, *v*/*v*/*v*) were efficient at yielding extracts with high total phenolic compound (TPC) and thiosulfinate (TS) content. According to the regression model computed, the most significant solvent mixture to obtain high TPC recovery from dried garlic powder was the binary mixture with 75% water and 25% acetone. For the obtention of high TS recovery from dried garlic powder, the most significant solvent was pure water (100%).

Both optimized extracts presented antioxidant activities, pure water being better in TAC and ORAC, and the binary mixture of water–acetone (75:25) being better in DPPH free radical scavenging activity.

Therefore, these optimized solvent mixtures can be used for industrial and research applications, providing extracts with a high number of bioactive compounds and, consequently, high antioxidant activities.

## Figures and Tables

**Table 1:** HPLC elution condition employed for constituent identification of *Allium sativum*.

Time (Min)	Water ^a^ (%)	Methanol ^b^ (%)
0	95	05
12	85	15
20	70	30

^a^ ultrapure water (acidified with 0.1% phosphoric acid); ^b^ HPLC grade methanol (acidified with 0.1% phosphoric acid).

**Table 2:** Total phenolic compound (TPC) and thiosulfinate (TS) content of the different solvent mixtures extracts.

Run	Water	Ethanol	Acetone	TPC (mg GAE ^a^ g^−1^ DW ^b^)	TS (μmol g^−1^ DW)
1	1	0	0	3.82 ± 0.07	6.42 ± 0.22
2	0	1	0	0.84 ± 0.01	1.40 ± 0.09
3	0	0	1	0.35 ± 0.02	1.43 ± 0.18
4	1/2	0	1/2	4.28 ± 0.12	3.41 ± 0.15
5	1/2	1/2	0	3.62 ± 0.08	2.78 ± 0.25
6	0	1/2	1/2	0.26 ± 0.01	0.33 ± 0.13
7	1/3	1/3	1/3	3.20 ± 0.04	2.11 ± 0.09
8	1/6	1/6	2/3	1.71 ± 0.07	1.18 ± 0.16
9	1/6	2/3	1/6	2.60 ± 0.08	1.53 ± 0.05
10	2/3	1/6	1/6	5.84 ± 0.03	3.69 ± 0.31

^a^ GAE—gallic acid equivalents; ^b^ DW—dry weight.

**Table 3:** Results of the analysis of variance (ANOVA) presenting the fitness of the cubic model estimated from simplex axial design for predicting the behavior of total phenolic compounds (TPC) and thiosulfinates (TS) for solvent optimization.

	Variation Source	Sum of Squares	Degrees of Freedom	Mean Square	Calculated F-Value	Probability
**TPC**	Model	64.73069	8	8.091336	1606.606	0.000000
	Total Error	0.10576	21	0.005036		
	Lack of Fit	0.01448	1	0.014477	3.172	0.090111
	Pure Error	0.09128	20	0.004564		
	Total Adjusted	64.83645	29	2.235740		
**TS**	Model	82.58388	8	10.32299	268.4487	0.000000
	Total Error	0.80754	21	0.03845		
	Lack of Fit	0.12249	1	0.12249	3.5761	0.073193
	Pure Error	0.68505	20	0.03425		
	Total Adjusted	83.39142	29	2.87557		

**Table 4:** Antioxidant activities of garlic extracts optimized for higher contents of thiosulfinates (100% water) and phenolic compounds (75% water and 25% acetone).

Water (%)	Ethanol (%)	Acetone (%)	TAC ^a^ (mg AAE ^d^ g^−1^ DW ^e^)	DPPH IC_50_^b^ (mg mL^−1^)	ORAC ^c^ (µmol TE ^f^ g^−1^ DW)
100	0	0	200.31 ± 0.93 a *	3.40 ± 0.35 b	882.47 ± 141.04 a
75	0	25	171.30 ± 2.13 b	2.88 ± 0.26 a	794.63 ± 135.99 a

* means followed by the same letter in columns do not differ statistically by Tukey’s test (*p* < 0.05). ^a^ TAC—total antioxidant capacity; ^b^ DPPH IC_50_—sample concentration in which the free radical scavenging activity percentage reaches 50%; ^c^ ORAC—oxygen radical absorbance capacity; ^d^ AAE—ascorbic acid equivalent; ^e^ dry weight; ^f^ TE—Trolox equivalent.

## Data Availability

Not applicable.

## Sample Availability

Not available.
